# Screening canine sources for novel antimicrobials reveals the circular broad-spectrum bacteriocin, caledonicin, produced by *Staphylococcus caledonicus*

**DOI:** 10.3389/fmicb.2024.1470988

**Published:** 2024-08-26

**Authors:** Michelle O’Connor, Paula M. O’Connor, David Hourigan, Ellen Murray, Felipe Miceli de Farias, Des Field, Colin Hill, R. Paul Ross

**Affiliations:** 1 School of Microbiology, University College Cork, Cork, Ireland; 2 APC Microbiome Ireland, University College Cork, Cork, Ireland; 3 Teagasc Food Research Centre, Moorepark, Fermoy, Cork, Ireland

**Keywords:** antimicrobial resistance, canine microbiota, bacteriocins, canine sources, antimicrobial potential, antimicrobial peptides

## Abstract

**Introduction:**

Antimicrobial-resistant pathogens present an ongoing threat to human and animal health, with deaths linked to antimicrobial resistance (AMR) predicted to increase annually. While the misuse and overuse of antibiotics in humans undoubtedly contribute to this escalation, antibiotic use in the veterinary field, including companion animals, also plays a contributing role. Pet owners’ desire to improve the quality of life of their pets is likely to support antibiotic use in this field. Consequently, there is a need for antibiotic alternatives to treat bacterial infections. This study set out to screen for antimicrobial peptides known as bacteriocins from bacterial isolates of aerobic/microaerophilic environments of canine sources and determine their potential as antibiotic alternatives against clinically relevant pathogens.

**Methods:**

Following a laboratory-based protocol, 22 bacterial isolates were subjected to whole-genome sequencing (WGS), and a total of 14 putative novel bacteriocins were identified from both class I and II bacteriocin classes. One particular bacteriocin, herein named caledonicin, was identified via *in silico* analysis from a *Staphylococcus caledonicus* strain and partially purified for further *in vitro* evaluation.

**Results:**

Caledonicin is a 64-amino acid (IAANLGVSSGTAYS MANALNNISNVATA LTIIGTFTGVGTIGSGIA ATILAILKKKGVAAAAAF) novel circular bacteriocin most closely related to enterocin_NKR-5-3B based on core peptide alignment (39.1%), with a molecular weight of 6077.1 Da. Caledonicin exhibits a broad-spectrum of activity against a range of pathogenic bacteria, including methicillin-resistant *Staphylococcus aureus* (MRSA), methicillin-resistant *Staphylococcus pseudintermedius* (MRSP), and *Listeria monocytogenes*; and the gut-related bacterium associated with Crohn’s disease, *Mediterraneibacter gnavus* ATCC 29149 (previously *Ruminococcus gnavus* ATCC 29149).

**Discussion:**

This represents the first bacteriocin screening study involving bacteria from canine sources and confirms this is a rich environment for bacteriocin-producing strains. This study also identifies and characterises the first novel bacteriocin from the staphylococcal species, *Staphylococcus caledonicus*.

## Introduction

While AMR is a serious health concern for humans, it is also a problem in veterinary settings (Meade et al., 2020). A contributing factor in the development of resistance in animals is the misuse and/ or overuse of antibiotics, including their use for improved growth in livestock. It is reported that over 70% of all antibiotics (including those defined as medically important for the treatment of infections in humans by the Food and Drug Administration) are also sold for use in animals in the USA (O’Neill, 2019).

Unlike the many reports of the acquisition of antibiotic resistance genes (ARGs) in livestock associated with the use of antibiotics, there is a scarcity of such data in companion animals (Pomba et al., 2017; Rendle and Page, 2018). The spread of antibiotic-resistant bacteria between pets and humans can occur via contact with the animal, physical injuries (Caneschi et al., 2023), or transfer of genetic material from the owner’s residential microbiota to that of the pet, or vice versa (Rendle and Page, 2018). Although it appears the use of antibiotics in companion animals has a less significant impact on the development and spread of ARGs compared to that of humans and livestock based on sales data (Graham et al., 2019), this is still a sector of animal health that requires more attention (Caneschi et al., 2023). This includes a proposal for the development of new antimicrobial compounds for the treatment of antibiotic-resistant infections caused by pathogenic bacteria in the veterinary field (World Health Organization, 2021), including MRSA, MRSP, *Pseudomonas aeruginosa*, extended-spectrum beta-lactamase (ESBL) *Escherichia coli, Klebsiella pneumoniae*, and vancomycin-resistant *Enterococcus* sp. (VRE) (Palma et al., 2020; Caneschi et al., 2023).

One potential alternative treatment for these infections is bacteriocins, antimicrobial peptides produced by bacteria that target closely related species. Bacteriocins are divided into two classes based on the presence (Class I) or absence (Class II) of post-translational modifications (PTMs) (Soltani et al., 2021). Nisin is a class I lantibiotic and the most studied bacteriocin since its discovery in 1928. It has antibacterial activity against a range of pathogens, including veterinary-related organisms such as *Enterococcus faecium, Streptococcus agalactiae*, and *Staphylococcus aureus* (Field et al., 2023). There have been a number of nisin-based products formulated for the treatment of veterinary-associated infections. These include Ambicin® (Applied Microbiology, Inc., New York, NY), MastOut®, and WipeOut®(Immucell Corporation), which have been shown to be effective treatments of bovine mastitis caused by *S. aureus* in cows (Sears et al., 1992; Cotter et al., 2005; Cao et al., 2007). Bayer has also developed nisin-incorporated wipes (Preva® Medicated Wipes) containing 25 μg ml^−1^ nisin for topical use in dogs, cats, and horses (Field et al., 2015), highlighting the potential for bacteriocins instead of, or in combination with, antibiotics for therapeutic use in veterinary medicine.

Another class of bacteriocins are the circular bacteriocins, so named due to their N- to C-terminal covalent linkage resulting in the formation of a circular structure. These bacteriocins have been considered to belong to class IIc bacteriocins; however, suggestions have been made to reclassify these peptides to the modified class I due to the presence of this linkage (Alvarez-Sieiro et al., 2016; Pérez-Ramos et al., 2021; Sugrue et al., 2024). There are approximately 20 circular bacteriocins which have been identified to date, with enterocin-AS-48 as the representative bacteriocin for this class (Galvez Frontiers in Microbiology 02 frontiersin.org et al., 1986). Circular bacteriocins are gaining more attention in the bacteriocin field and can be identified via *in silico* analysis of bacterial genomes in high abundance, as shown by Xin et al. where nearly 7,000 putative circular prepeptide genes were identified across 86 species (Xin et al., 2020).

In this study, we screened bacteria isolated from canine sources for antimicrobial activity with the aim of finding and characterising putative novel bacteriocins that could aid in the fight against AMR. To the best of our knowledge, this is the first such study to search for putative bacteriocins from canine sources. One of 14 putative novel bacteriocins identified in this study via whole genome *in silico* analysis was a circular bacteriocin, similar to enterocin-NKR-5-3B. This bacteriocin was produced by a novel species of *Staphylococcus, Staphylococcus caledonicus*, that was first isolated and identified from a canine in Scotland in 2021 (Newstead et al., 2021). As a consequence of its novelty in terms of bacterial species, its canine-related source, and its circular nature, this bacteriocin, designated caledonicin, was selected for further characterisation. To that end, semi-purified preparations of caledonicin were obtained and subjected to an array of conventional bacteriocin assessment methods, including MALDI-TOF mass spectrometry, protease sensitivity, heat stability, and spectrum of inhibition assays against a variety of relevant bacterial indicator organisms. This is both the first bacteriocin to be reported and characterised from the *S. caledonicus* species and the first circular bacteriocin from the *Staphylococcus* genus to be confirmed via correlation of MALDI-TOF mass spectrometry of an active extract which exhibits antimicrobial activity against both veterinary- and human-related pathogens.

## Materials and methods

**Figure 1:**
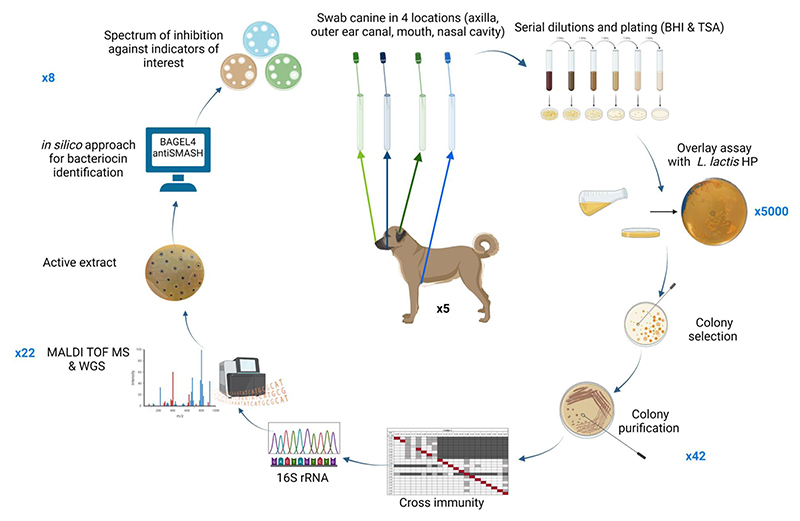
Schematic representation of methods performed in this screening study. Numbers in blue outside the circle represent the number of bacterial isolates brought forward at each stage of the screen. Figure created using BioRender.

A schematic representation of the methods performed in this study is presented in Figure 1.

### Isolation of antimicrobial isolates from canine sources

Five canines (four females and one male), ranging between 7 and 11 years of age at the time of swabbing, participated in this study. Four areas of the body were swabbed: the ear canal, the axillary vault (underarm), the gumline/teeth, and the inner nares (nostril). Each swab (Aptaca, sterile swabs; Aptaca S.p.A., Regione Monforte, 30-14053 Canelli, Italy) was dipped in sterile saline solution (PBS) before swabbing the site of interest, and swabs were stored for no more than 2 h at 4°C before 10-fold serial dilutions were performed in PBS and 100 μL aliquots were spread-plated onto Brain Heart Infusion (BHI) agar (Merck, Darmstadt, Germany) and Tryptic Soy Agar (TSA) (Merck Millipore). All plates were incubated aerobically at 37°C for 24−48 h.

### Detection of antimicrobial activity from bacterial isolates from canines

Deferred antagonism assays were performed for the detection of antimicrobial production by bacterial isolates from canine sources as follows. A 1% inoculum of indicator organism *Lactococcus lactis* subsp. *cremoris* HP (*L. lactis* HP) was sub-cultured overnight in 10 mL of M17 broth supplemented with glucose (GM17) (0.5% w/v) and incubated at 30°C overnight. Plates with bacterial isolates from canine samples grown as described above on BHI and TSA were overlaid with GM17 sloppy agar (0.75% w/v agar) seeded with 0.25% inoculum of *L. lactis* HP indicator strain and grown aerobically overnight at 30°C (see Table 1 for optimal growth conditions of the bacterial strains used in this study). Colonies from this assay that exhibited zones of inhibition were then inoculated into BHI broth, grown overnight at 37°C, and stocked in 40% glycerol at −80°C for further characterisation.

### Characterisation of antimicrobial-producing canine isolates

#### Cross-immunity

To determine the relatedness of bacteriocins produced by the antimicrobial-producing isolates, cross-immunity assays were performed using deferred antagonism assays as previously described. Briefly, 96-well microtitre plates with all antimicrobial-producing isolates were stamped (approximately 2 μL) onto BHI agar plates and incubated overnight at 37°C. The spots were then subjected to UV treatment in a CL-1000 Ultraviolet Crosslinker for 45 min. Following this, 20 mL volumes of sloppy BHI (at 0.75% w/v agar) were prepared and inoculated individually with 0.25% of an overnight culture of each antimicrobial-producing canine isolate. Plates were overlaid with the inoculated sloppy agar and incubated overnight at 37°C.

#### 16S rRNA sequencing

Colony PCR was performed on antimicrobial-producing strains. Cells were lysed in 25 μL of PCR-grade water via microwaving for 50 s and centrifuged at 11,500 × *g* for 1 min. PCR was performed in a total volume of 50 μL using 25 μL of MyTaq™ Mix PCR master mix (Bioline), 19 μL of PCR-grade water, 2 μL of the non-specific primers 27F (5′-AGAGTTTGATCATGGCTCA-3′) and 1492R (5′-TACGGTT ACCTTGTTACGACTT-3′) (primer stocks at 10 μM; Eurofin Genomics), and 2 μL of DNA template from lysed cells. Amplification was carried out with reaction conditions as follows: initial denaturation at 95°C for 1 min, followed by 35 cycles of 95°C for 15 s, annealing at 55°C for 15 s, and elongation at 72°C for 10 s with a final extension step at 72°C for 10 min. Five μl of the resulting amplicons from each reaction were electrophoresed in a 1.5% (w/v) agarose gel. An OmegaFlour Plus™ Gel Documentation System (Aplegen, San Francisco, United States) was used for visualisation. The PCR products were purified using the GeneJet PCR Purification Kit (Thermo Fisher Scientific, Waltham, MA, United States). DNA sequencing of the forward strand was performed by Genewiz (Leipzig, Germany). The resulting sequences were identified to species level (≥98%) using BLAST for comparison to sequences deposited in the GenBank database.

#### Whole-genome sequencing and *in silico* mining for bacteriocin operons

DNA from 22 antimicrobial-producing isolates selected for whole-genome sequencing was extracted by growing cultures overnight in 10 mL BHI broth at 37°C. Genomic DNA was extracted using a Sigma-Aldrich DNA purification kit as described by the manufacturer (Sigma-Aldrich Ireland Limited, Vale Road, Arklow, Co. Wicklow, Ireland). A total of 22 genomes were subjected to WGS, of which 12 (APC 4160, 4164, 4166, 4149, 4130, 4133, 4148, 4170, 4140, 4157, 4158, and 4171) were sequenced with the Illumina MiSeq Sequencing System by GenProBio (University of Parma). The remaining 10 genomes (APC 4161, 4163, 4136, 4156, 4152, 4153, 4147, 4137, 4145, and 4154) were sequenced via the Illumina sequencing platform with MicrobesNG (Birmingham, United Kingdom). WGS was downloaded in FASTQ format. Quality control was run on the reads using FastQC v0.11.9 and Fastp v0.23.2. The genomes were assembled using Spades v3.15.5. Quality control was run on the genomes using CheckM2 v1.01. Genome assemblies were annotated using Bakta v1.8.1 and database v5. *In silico* bacteriocin mining tools BAGEL4 (Van Heel et al., 2018) and antiSMASH7.0 (Blin et al., 2023) and the genome visualisation tool Artemis (Carver et al., 2012) were used for the analysis of genome sequences to identify putative novel bacteriocin operons. To determine the level of novelty of bacteriocins identified and to compare these operons to existing bacteriocins against the National Centre for Biotechnology Information (NCBI) database, the BLASTp (Protein Basic Local Search Tool) tool was used. Amino acid sequences of putative novel bacteriocins predicted in whole-genome sequenced isolates were aligned with closely related bacteriocin leader and core peptides using the Multiple Sequence Alignment (MSA) tool MUSCLE, accessed via EMBL-EBI’s job dispatcher (Madeira et al., 2022) and then visualised using Jalview (Waterhouse et al., 2009), where pairwise alignment was performed, and percentage identity was calculated to determine the most closely related known bacteriocin to the ones found in this study. It should be noted that MSA was only carried out on circular bacteriocin, lasso peptide, and lanthipeptide classes of bacteriocins, which are the main focus of our research group. Although novel bacteriocin operons belonging to the thiopeptide/thiazole/oxazole-modified microcins (TOMMs) class were identified in the course of this study, they were not examined in detail but may be the subject of more focused investigation in the future studies. Bacteriocins were considered novel when one or more amino acid changes in their core peptide were observed compared to their most closely related bacteriocin.

#### MALDI-TOF mass spectrometry

MALDI-TOF mass spectrometry was performed on colonies from the 22 antimicrobial-producing isolates subjected to whole-genome sequencing (data not shown) and a partially purified 70% IPA fraction of caledonicin. Briefly, colonies were mixed with 50 μL propan-2-ol 0.1% TFA, vortexed three times, and centrifuged at 16,000 × *g* for 30 s. MALDI-TOF mass spectrometry was performed on the cell-free supernatant (CFS) using an iDPlus Performance MALDI-TOF mass spectrometer (Shimadzu Europa GmbH, Duisburg, Germany). An aliquot (0.5 μL) of matrix solution (α-cyano 4-hydroxy cinnamic acid, 10 mg ml^−1^ in acetonitrile (0.1%; v/v) trifluoroacetic acid) was deposited onto the target and left for 20 s before being removed. The residual solution was allowed to air-dry, and 0.5 μL of the sample solution was deposited onto the pre-coated sample spot. 0.5 μL of matrix solution was added to the deposited sample and allowed to air-dry. Samples were then analysed in positive-ion linear mode.

#### Spectrum of inhibition of putative novel bacteriocin producers

Deferred antagonism assays to determine the spectrum of inhibition of the eight isolates harbouring putative novel bacteriocin operons were conducted as previously described, with minor modifications. Approximately 2 μL of an overnight culture was spotted on BHI agar (1.5%) plates via a 96-well plate replicator (Boekel, Germany) before incubating aerobically at 37°C overnight. Following incubation, spots were chloroform-treated for 30 min before overlaying with eight gram-positive and seven gram-negative indicator strains as described above. Indicator strains employed to detect antimicrobial activity and their growth conditions are listed in Table 1A. Zones of inhibition, indicative of antimicrobial activity, were measured using Vernier callipers (resolution 0.05), recorded in millimetres (mm), and rounded to one decimal place. Activity was calculated as follows: diameter of zone minus diameter of colony in millimetres and expressed as the average between two.

### Characterisation of a novel circular bacteriocin, caledonicin, from *Staphylococcus caledonicus* APC4137

#### Assessment of bacteriocin production in *Staphylococcus caledonicus* APC4137 extracts

*S. caledonicus* APC 4137 extracts, including CFS, a 70% IPA 0.1% TFA cell extract, and freeze−thaw liquid (FT) from the agar on which the strain was grown, were tested against *L. lactis* HP and *Micrococcus luteus* (*M. luteus*) APC 4061 for activity via WDA. Briefly, M17 supplemented with 0.5% glucose and BHI agar was seeded with a 1% inoculum of the indicators *L. lactis* HP and *M. luteus* APC 4061, respectively. Wells were bored into the agar plates, and 50 μL of each extract was inoculated into the wells, followed by incubation overnight at temperatures relevant to the indicators tested (Table 1). Following incubation, wells were checked for zones of inhibition.

#### Partial purification of caledonicin from *Staphylococcus caledonicus* APC 4137

Freeze−thaw liquid for semi-purification of caledonicin was prepared as follows. *S. caledonicus* APC 4137 was grown overnight in BHI at 37°C and 500 μL of this overnight culture was spread-plated on BHI sloppy agar (0.75%) plates before incubating overnight at 37°C. Following incubation, the plates were then overlaid with GM17 sloppy agar and inoculated with a 0.25% inoculum of *L. lactis* HP before incubating overnight at 30°C. Following incubation and observation of antimicrobial activity of the indicator overlay, the plates were frozen at −80°C for 1 h, followed by thawing at room temperature. FT liquid from thawed plates was collected, filter sterilised through a 0.2 μm filter, and subsequently passed through a 10-g (60 mL) Strata C18-E SPE column (Phenomenex) pre-equilibrated with 60-ml methanol (Fisher Scientific, United Kingdom) and 60 ml HPLC-grade H_2_O. After 60 mL of 30% ethanol was applied, the antimicrobial was eluted from the column using 60 mL of 50, 60, and 70% isopropanol. Eluted fractions were tested for antimicrobial activity against *L. lactis* HP via WDA. Samples were also subjected to MALDI-TOF mass spectrometry (described above) to determine the presence of the corresponding mass of the circular bacteriocin.

#### Heat stability and protease sensitivity of caledonicin from *Staphylococcus caledonicus* APC 4137

The heat stability and protease sensitivity of caledonicin were determined by WDA on the semi-purified C18 fraction eluted in 70% IPA, following confirmation of the predicted mass by MALDI-TOF mass spectrometry. The C18 70% eluent was incubated for 15 min at a range of temperatures: 37°C, 65°C, 80°C, 100°C, and 120°C, before performing WDA in 1.5% M17 agar supplemented with 0.5% glucose and seeded with 0.25% of *L. lactis* HP. Untreated C18 70% eluent was used as a control. To investigate if the antimicrobial produced was proteinaceous in nature, the eluent was subjected to protease treatment with proteinase K (Sigma-Aldrich) to a final concentration of 20 mg ml^−1^ at 37°C for 3 h before heat deactivating the protease enzyme at 95°C for 5 min and testing for antimicrobial activity via WDA as described above. Controls for this test included untreated caledonicin containing eluent (diluted to the same concentration as test caledonicin containing eluent) and heat-treated caledonicin containing eluent (95°C for 5 min) without protease treatment. To determine the specific protease sensitivity of caledonicin, the semi-purified fraction was treated with trypsin, α-chymotrypsin, pepsin, and proteinase K (Sigma-Aldrich) to a final concentration of 100 μg ml^−1^ and incubated at 37°C for 3 h. Enzymes were deactivated with heat treatment as above before testing for antimicrobial activity against *L. lactis* HP via WDA.

#### Antimicrobial activity spectrum of caledonicin

Following confirmation of activity of the semi-purified caledonicin preparation against *L. lactis* HP and the presence of the correct predicted molecular mass, the spectrum of inhibition of caledonicin was determined via WDA against the eight gram-positive indicators used in overlay assays for all novel bacteriocin producers in this study (Table 1A); and six other gram positive indicators. Four of these six partially make up a Simplified Human Intestinal Microbiota (SIHUMI) consortium (Ríos Colombo et al., 2023) This simplified gut consortium included Enterococcus faecalis OG1RF, Lactiplantibacillus plantarum WCFS1, Bifidobacterium longum ATCC 15707 and Mediterraneibacter gnavus ATCC 29149 with the addition of Clostridioides difficile APC43 indicator (Table 1B). These strains were grown at 37 °C under strict anaerobic conditions (Type A vinyl anaerobic chamber, Coy Labs) in a modified Brain Heart Infusion (Oxoid) medium with the addition of 0.5% yeast extract, 5 mg/L hemin (Sigma−Aldrich), 1 mg/ml cellobiose (Sigma−Aldrich), 1 mg/ml maltose (Sigma−Aldrich), and 0.5 mg/ml L-cysteine (Sigma−Aldrich) − LYHBHI. Lactobacillus delbrueckii subsp. bulgaricus LMG6901 was also used as an indicator in this assay, and grown anaerobically in MRS media at 37 °C (Table 1B).

#### Structural prediction of caledonicin

Structural characterisation of the circular bacteriocin from *S. caledonicus* APC4137 was performed via the online tool ColabFold v1.5.5: AlphaFold2, followed by visualisation of the bacteriocin in the PyMOL Molecular Graphics System, v 3.0 (Schrödinger, LLC) with an extension prompt to colour structures from the AlphaFold Protein Structure Database by predicted local distance difference test (pLDDT), where dark blue regions indicate high confidence (pLDDT >90).

## Results

### Antimicrobial potential from canine sources

#### Cross-immunity of antimicrobial-producing bacterial isolates from canines

**Figure 2:**
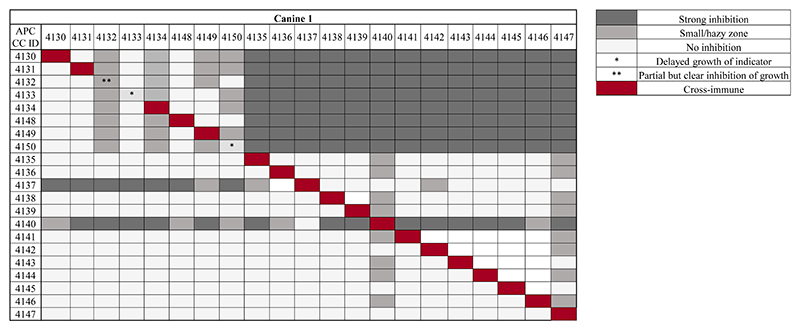
Cross-immunity of antimicrobial-producing isolates from one of five canines in this study using overlay assays. Strains listed in the top row are indicators overlaid against antimicrobial-producing strains listed on the left column of the table. Strength of inhibition by the antimicrobials produced is indicated in the key (right), where the darker the colour grey, the stronger the inhibition observed. Red diagonal boxes indicate the producer is immune to its own antimicrobial, with the exception of three bacterial isolates for this particular canine, marked with asterisk(s) (*/**). One asterisk (*) represents producers whose antimicrobial delays growth when tested against itself, while two asterisks are representative of the producer exhibiting definite inhibition of growth when tested against itself (**). Cross-immunity of all other canines in this study is available in Supplementary Table S1.

Based on the presence of zones of inhibition against the indicator *L. lactis* HP in agar overlay assays, 42 antimicrobial producers were isolated from approximately 5,000 colonies screened from the five canines in this study. Cross-immunity tests were performed for isolates from each dog against each other isolate. All producers were immune to the activity of their own antimicrobial(s), with the exception of four strains (APC 4132, APC 4133, and APC 4150 from canine 1 and APC 4160 from canine 3) (Figure 2; Supplementary Table S1). Isolates selected for further testing were based on this assay. Isolates APC 4130−4134 and APC 4148−4150 from canine 1 all exhibit a similar trend in activity, indicating the probability that these isolates are the same strain or are producing the same antimicrobial (Figure 2). This was supported by 16S rRNA sequencing at the species level, where all of these isolates were identified as *Bacillus cereus*. Isolates APC 4137 and APC 4140 exhibit completely different activity profiles compared to all other isolates from that same canine and were therefore selected for further characterisation. Isolates APC 4137 and APC 4140 were confirmed by whole-genome sequencing to be *S. caledonicus* and *Bacillus pumilus*, respectively. Although some isolates, such as *B. cereus* APC 4147 and APC 4145, exhibited little activity against indicators in this assay, they did inhibit the growth of the indicator *L. lactis* HP in the original overlay assays. Therefore, the isolates brought forward for further analysis were those determined by the results of both cross-immunity and those with activity in the initially deferred antagonism assays against *L. lactis* HP. These methods of initial characterisation and the basis for bringing these specific isolates of interest forward in this study could be considered a limitation due to the use of only one indicator in the initial screening process, as well as the limited number of media used for bacterial growth. Cross-immunity was also performed for the isolates from all other canines in this study (Supplementary Table S1).

#### 16S rRNA sequencing

16S rRNA sequencing of antimicrobial-producing bacterial isolates revealed a total of eight different species across the 42 isolates sequenced. The most abundant genus isolated in this study was *Staphylococcus* (*n* = 24), followed by *Bacillus* (*n* = 15), *Actinomyces* (*n* = 2), and *Paenibacillus* (*n* = 1).

#### Bacteriocin identification via *in silico* mining of whole genomes and AMR/virulence gene detection

The 22 genomes subjected to whole-genome sequencing represent a range of antimicrobial-producing species, including *B. cereus* (*n* = 7), *S. pseudintermedius* (*n* = 7), *S. caledonicus* (*n* = 2), *S. warneri* (*n* = 1), *Actinomyces bowdenii* (*n* = 2), *Bacillus pumilus* (*n* = 1), *Bacillus safensis* (*n* = 1), and *Paenibacillus* sp. (*n* = 1). These strains were selected based on the location of the canine where the organism was isolated, colony morphology, cross-immunity profile, and activity against *L. lactis* HP in original overlay assays. There were multiple bacteriocin operons from Classes I and II within these 22 genomes, as identified by BAGEL4 and antiSMASH7.0 (Table 2). Of these 22 genomes, eight were selected for further analysis based on other factors. These include the initial antimicrobial profiles observed when genomes were put through the RiPP and secondary metabolite software tools, the average nucleotide identity (ANI) of genomes of the same species (calculated by EZBioCloud)^1^ (data not shown), and MALDI-TOF mass spectrometry profiles (data not shown). The genomes of these eight bacterial isolates were further studied for novel bacteriocin operons by *in silico* analysis with BAGEL4 and antiSMASH7.0 and comparison to closely related bacteriocins based on BLAST results and sequence alignment. Within the eight isolates, a total of 14 putative novel bacteriocins were identified, which are presented in Table 3 with their predicted leader and core amino acid sequences, predicted mass, and the online tool utilised, if applicable.

While this study aimed to find putative novel bacteriocins from canine sources, it was noted that some of the antimicrobial-producing organisms were of the same pathogenic species associated with animal health from an AMR perspective, e.g., *Staphylococcus pseudintermedius*. Therefore, all genomes sequenced in this study were tested for the presence of antimicrobial resistance and virulence genes using the Comprehensive Antibiotic Resistance Database (CARD) and Virulence Factor Database (VFDB), respectively, with set parameters of 80% identity and coverage through the ABRicate software tool on Galaxy (version 23.1.rc1) (Supplementary Table S2). Briefly, 12 of the 22 genomes encoded resistance to penam antibiotics due to the presence of the genes BcI/BcII (*B. cereus*), BPU-1 (*B. safensis*), *blaZ* (*S. caledonicus*), and *mgrA* (*S. caledonicus* and *S. warneri*). Resistances to cephalosporins and macrolide antibiotics were the second most abundant class, with 10 isolates encoding resistance based on the presence of *mefE, ermA, mphC*, and *msrA* genes. The remainder of ARGs reported and the number of isolates with resistance determinants were glycopeptides and fosfomycin (*n* = 7 each), acridine dyes, fluoroquinolones, peptide antibiotics, tetracyclines, and lincosamides (*n* = 3), and fusidic acid and rifamycin (*n* = 1). Interestingly, no ARGs or virulence factors could be found in the two genomes of *A. bowdenii* (APC 4154 and APC 4158), as well as the *B. pumilus* isolate (APC 4140), based on the parameters set in the software tools used. Virulence genes detected using the VFDB software revealed nine virulence genes translating to the following virulence factors: metalloproteases, non-haemolytic enterotoxin A, B, and C, haemolysin BL-binding component precursor, haemolysin BL lytic component L1 and L2, cytotoxin K, and thiol-activated cytolysin. These virulence genes were only detected in the genomes of *Bacillus cereus* isolates. Based on these results, it is unlikely that these antimicrobial-producing strains could be used as probiotics, other than *A. bowdenii* (APC 4154) and *B. pumilus* (APC 4140). The AMR and virulence genes detected are summarised in Supplementary Table S2.

#### Spectrum of inhibition of putative novel bacteriocin-producing isolates

A total of eight antimicrobial-producing isolates from this study were tested against eight gram-positive and seven gram-negative indicators of interest (Table 1). The producers include *S. pseudintermedius, S. warneri, S. caledonicus, A. bowdenii, B. cereus, B. safensis, B. pumilus*, and *Paenibacillus* sp. Although *L. lactis* HP was sensitive to all of the bacteriocin producers (consistent with their isolation against this indicator), there was also activity observed against multidrug-resistant (MDR) relevant veterinary pathogens. Of the *Staphylococcus* species identified, *S. pseudintermedius* APC 4170 displayed the greatest spectrum of activity against the indicators tested (5/15); however, the zone of inhibition produced by *S. caledonicus* APC 4137 against the indicators *L. lactis* HP and *L. innocua* UCC was larger than that of *S. pseudintermedius* APC 4170 and *S. warneri* APC 4145. *S. warneri* APC 4145 only displayed activity against two of the indicators, *M. luteus* APC 4061 and *L. lactis* HP. *A. bowdenii* APC 4154 displayed activity against all eight gram-positive indicators. While the greatest activity observed was against *L. lactis* HP, there was also activity against MDR pathogens tested (Table 4). This, in combination with the absence of antibiotic resistance genes and virulence factors (Supplementary Table S2), within this isolate could be a basis for further testing of the antimicrobial(s) produced and the potential for this strain as a probiotic. Of the *Paenibacillus/Bacillus* isolates, *B. cereus* APC 4133 inhibited the growth of all eight gram-positive indicators tested, while *B. safensis* APC 4157 and *B. pumilus* APC 4140 displayed activity against seven and five of the gram-positive and two and one of the gram-negative indicators, respectively. The *Paenibacillus* isolate also inhibited the growth of all indicators tested. This was not unexpected given the fact that this genus is known to produce multiple antimicrobial compounds, including polymyxins and lanthipeptides (Li et al., 2021). All indicators tested in this assay are listed in Table 1A.

#### Multiple sequence alignment of novel bacteriocins and operon alignment

Multiple sequence alignment (MSA) of putative novel bacteriocins (Table 3) against known bacteriocins from the same class/subclass was carried out with MUSCLE, and alignments were viewed on Jalview, where the percentage identity of bacteriocins was performed via pairwise alignment. The MSAs reported in this study are grouped based on the genus from which the bacteriocins were identified.

**Figure 3:**
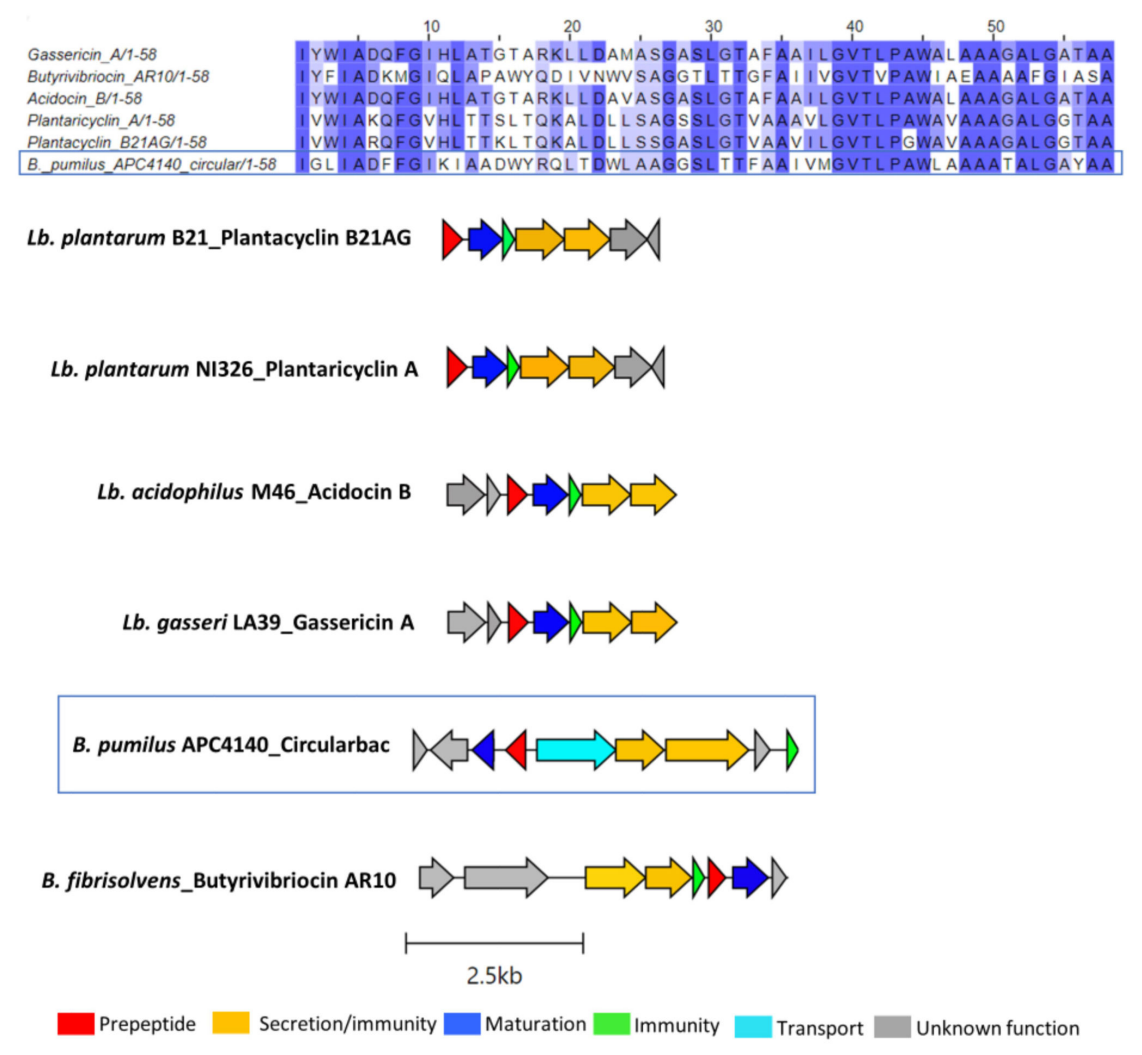
Multiple sequence alignments (MSA) of the circular bacteriocin core peptide identified within *B. pumilus* APC 4140 with subgroup II circular bacteriocins and gene operon alignment for the same. GenBank accession for class IIc bacteriocins in these alignments: Butyriovybriocin_AR10; AF076529.1, Gassericin A; AB007043.2, Acidocin B; KP728900.1, plantacyclin B21AG; CP025732.1, plantaricyclin A; NDXC01000075.1.

In total, seven putative novel bacteriocins were identified within the genus *Bacillus*. These include two circular bacteriocins (*B. pumilus* and *B. safensis*), two lanthipeptides (*Paenibacillus* sp.), and one lasso peptide (*Paenibacillus* sp.). The circular bacteriocin from *B. pumilus* APC 4140, while annotated as butyrivibriocin_AR10 in BAGEL4, was in fact most closely related to gassericin A and acidocin B, with 56.9% identity for both when pairwise alignment of the core peptides was calculated. The percentage identity when aligned with butyrivibriocin_AR10 and plantaricyclin A was 51.72% for both peptides and 46.55% when aligned with plantacyclin B21AG. An alignment of these six bacteriocin operons shows the presence of all genes required to translate to proteins responsible for circular bacteriocin synthesis (core peptide, sporulation M protein, and ABC transporters) with the exception of one extra transport-related gene within the *B. pumilus* APC 4140 operon, annotated as RND efflux transporter; therefore, it could be a potential other transport-related protein for the bacteriocin (Figure 3).

**Figure 4:**
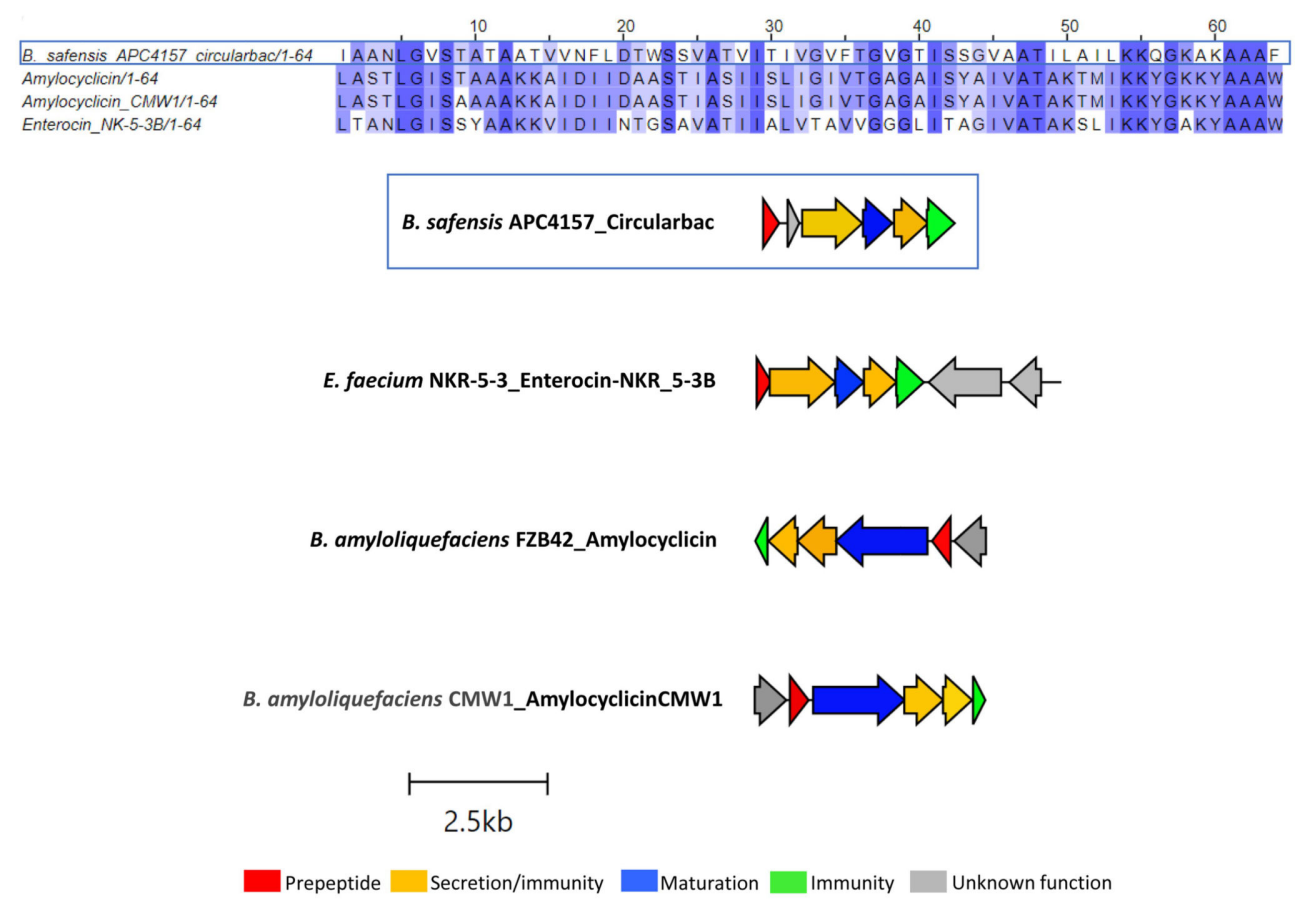
MSA of circular bacteriocin core peptide identified within *B. safensis* APC 4157 with subgroup II circular bacteriocins and gene operon alignment for the same. GenBank accession for class IIc bacteriocins in these alignments: Amylocyclicin; GCA_000015785.2, Amylocyclicin CMW1; GCA_000747705.1, Enterocin-NKR-5-3B; LC068607.1.

Another novel circular bacteriocin was found within the *B. safensis* APC 4157 genome by both BAGEL4 and antiSMASH7.0, with two core peptide genes identified. As this was the only circular bacteriocin identified in this study to contain two putative structural genes, the structure of the peptides was predicted using ColabFold v1.5.5: AlphaFold2. The product of the first structural gene was found to have a circular shape with 4 alpha-helix domains (consistent with circular bacteriocins), while the second gene product did not yield a circular structure. Due to this and no prior reports of two peptide circular bacteriocin operons, we assigned this gene as having an unknown function (Figure 4). Again, the most closely related bacteriocin was identified via pairwise alignment of core peptides with known circular bacteriocins, and amylocyclicin (40.6%), enterocin-NKR-5-3B (40.6%), and amylocyclicin CMW1 (39.1%) were found to be the most closely related. Whole operon alignments revealed that the bacteriocin operon of APC 4157 matches most closely with enterocin-NKR-5-3B, due to the presence of similar-sized maturation (blue) and ABC transporter-related proteins (Figure 4).

**Figure 5:**
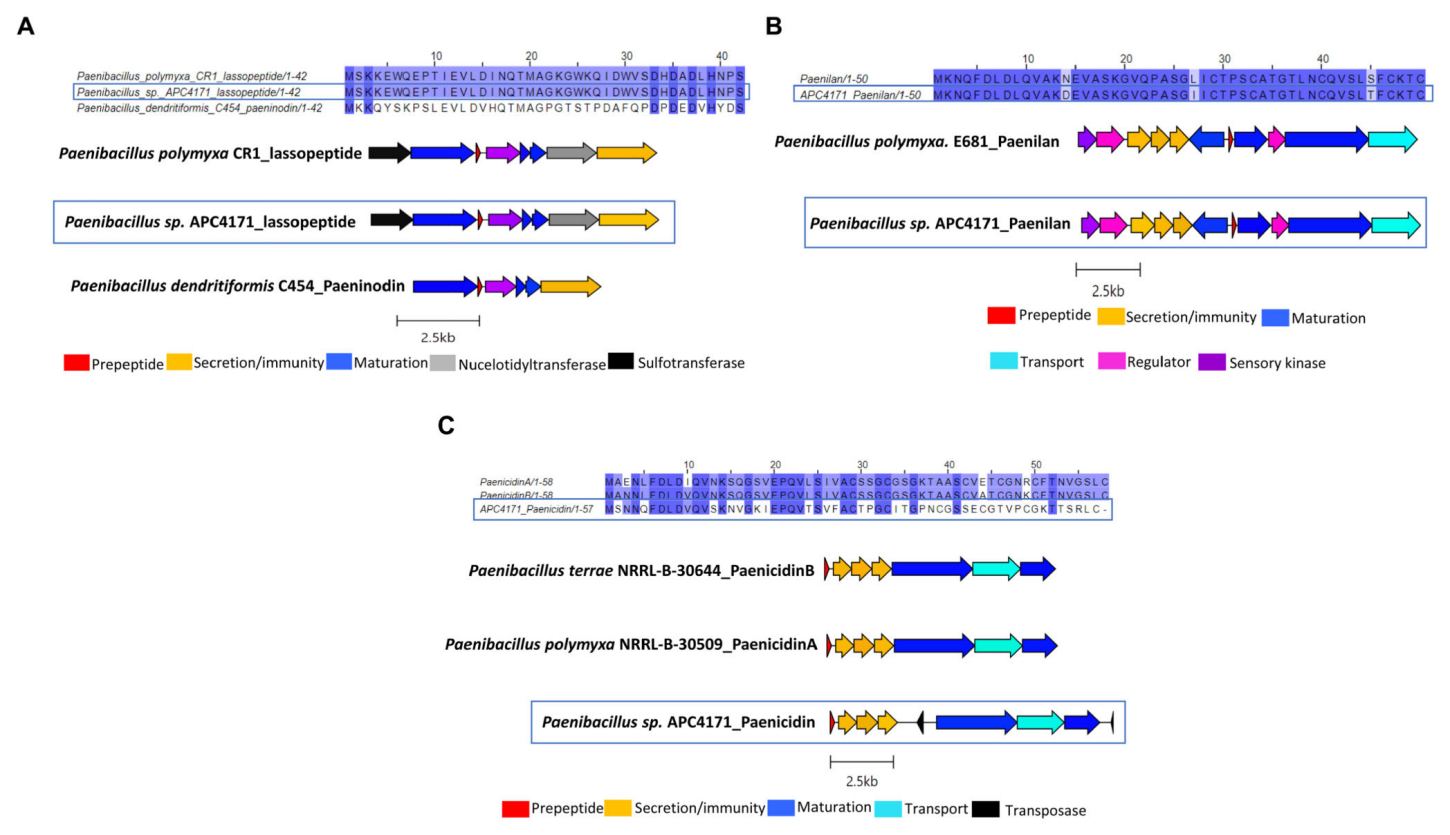
**(A)** MSA of lasso peptide identified within APC 4171 with paeninodin (Genbank accession; GCA_000245555.2) and another lasso peptide identified within another Paenibacillus species (CR1) (Genbank accession; GCA_000507205.2), and operon alignment of same. **(B)** MSA of lanthipeptide identified within APC 4171 with lanthipeptide paenilan (Genbank accession; CP000154) and gene operon alignment of same. **(C)** MSA of lanthipeptide identified within Paenibacillus sp. APC4171, with paenicidin A (Genbank accession; JTHO01000011.1) & paenicidin B (Genbank accession; JTHP01000001.1) and operon alignment for the same.

*Paenibacillus* sp. APC 4171 *in silico* analysis identified three putative novel bacteriocins, including two lanthipeptides and one lasso peptide. The lasso peptide was annotated as belonging to the paeninodin family of lasso peptides by BLASTp and therefore was aligned with this peptide (leader and core). However, following identification of the bacteriocin operon, the presence of genes encoding nucleotidyltransferase and sulfotransferase was identified in the bacteriocin operon. This was also reported by Zhu et al. in *Paenibacillus polymyxa* CR1 (Zhu et al., 2016). Due to the 100% amino acid identity between the core peptide of this strain and APC 4171, the bacteriocin operon from strains CR1 and C454 was both used for operon alignment with APC 4171 (Figure 5A). Briefly, pairwise alignment of the prepeptide (leader and core) paeninodin from strains C454 and APC 4150 was calculated at 45.2%, while alignment between APC 4171 and CR1 lasso peptides was 100%. Operon alignment demonstrates all three contain the relative genes required for biosynthesis of the lasso peptide, including maturation enzymes for relevant modifications (Figure 5A).

Two lanthipeptide operons were also identified within *Paenibacillus* sp. APC4171, one closely related to paenilan and the second most closely related to paenicidin B based on antiSMASH. Following the same procedures used for all other bacteriocins in this study, sequence alignments of the prepeptides (core and leader) with their closest relatives were carried out, and percentage identities via pairwise alignments were calculated. The paenilan-like lanthipeptide was found to be 94% identical to paenilan from *Paenibacillus polymyxa* E681, with three amino acid differences across the entire prepeptide (one residue difference in the leader and two in the core peptide). Operon alignments with the paenilan bacteriocin operon revealed that APC 4171 harbours all genes required for the synthesis of the lanthipeptide paenilan, including *lanB* and *lanC* encoding the maturation enzymes (Figure 5B). The second lanthipeptide identified in APC 4171 was recognised as having 100% similarity to paenicidin B as determined by antiSMASH, but when aligned with both paenicidin A and B, the percentage identity of these prepeptide sequences in comparison to the lanthipeptide in APC 4171 was only 44.1 and 47.5% identity to these peptides, respectively. Operon alignment again showed the lanthipeptide from APC 4171 contained all relevant genes for biosynthesis, including *lanB* and *lanC*. The presence of two transposase genes was also noted, one upstream and the other downstream of *lanBC* (Figure 5C).

**Figure 6:**
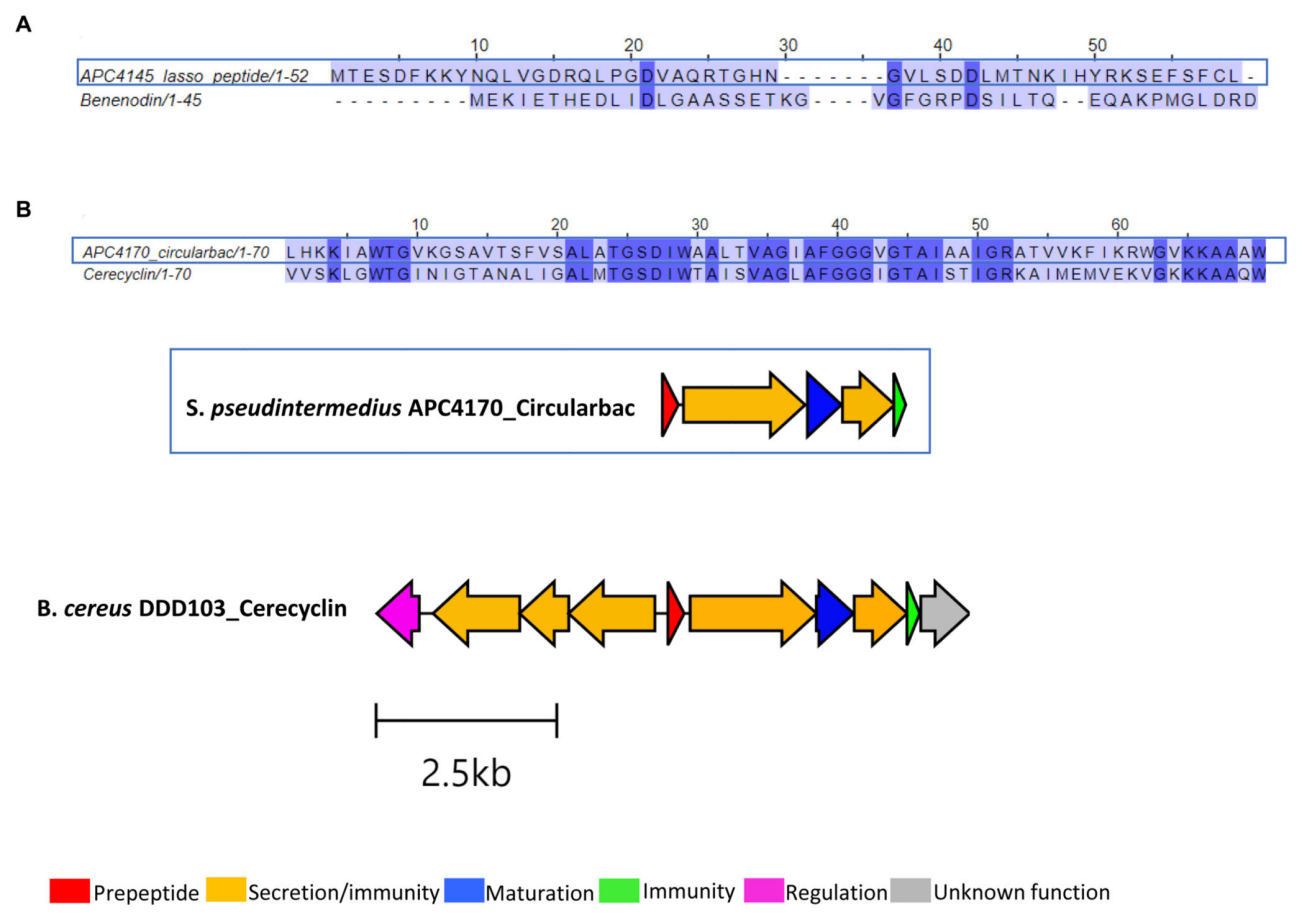
Sequence alignments of bacteriocin peptides identified within **(A)** *S. warneri* APC 4145 and **(B)** *S. pseudintermedius* APC 4170, and the biosynthetic operon for this circular bacteriocin aligned with the most closely related, cerecyclin (GenBank accession: MH037333.1). Only core peptide is aligned for the circular bacteriocin identified in APC 4170. Gene operon alignment was carried out for this same bacteriocin, but not for APC 4145.

Three putative novel bacteriocins were identified based on the presence of a prepeptide gene from the three different *Staphylococcus* species, two of which were circular bacteriocins and one lasso peptide. While the lasso peptide was annotated as a benenodin-family lasso peptide, its operon did not have the typical makeup of a lasso peptide gene operon, particularly in the absence of a gene encoding the cyclase involved in macrolactam ring formation. Therefore, this bacteriocin was not investigated further following sequence alignment with benenodin-1 (calculated as 20%) (Figure 6A). The circular bacteriocin identified from *S. pseudintermedius* APC 4170 harbours a predicted 70-amino acid core peptide (Figure 6B; Table 3). Following MSA with the core peptides of all known circular bacteriocins, the most closely related peptide to this novel peptide was cerecyclin, also a 70-amino acid core peptide but with four amino acids comprising the leader sequence. The pairwise alignment of these two core peptides was calculated at 48.6%. Gene operon comparison demonstrated that the operon from APC 4170 harbours the five genes typically found within circular bacteriocin operons. These include a gene encoding the prepeptide, two genes correlating to an ATP-binding protein and transporter permease (ABC transporter) with predicted function reported for secretion/immunity, a second, smaller, hydrophobic putative immunity protein gene, and most importantly, the putative maturation enzyme gene (sporulation M protein) for cyclisation of the N to C terminus or the core peptide. Although the cerecyclin operon is reported to contain a transcriptional regulator, this was not observed within the operon for APC 4170 (Figure 6B). The third putative novel bacteriocin from *Staphylococcus* is a circular bacteriocin from *S. caledonicus* APC 4137, which was selected for more in-depth characterisation and these results are described in more detail below.

It is worth noting that in this study, circular bacteriocins were aligned with core peptides only, due to the varying length of leader sequences across all circular bacteriocins (between 2 and 48 amino acids) (Perez et al., 2018).

### *In silico* analysis, partial purification, and characterisation of caledonicin from *Staphylococcus caledonicus* APC 4137

#### *In silico* analysis

**Figure 7:**
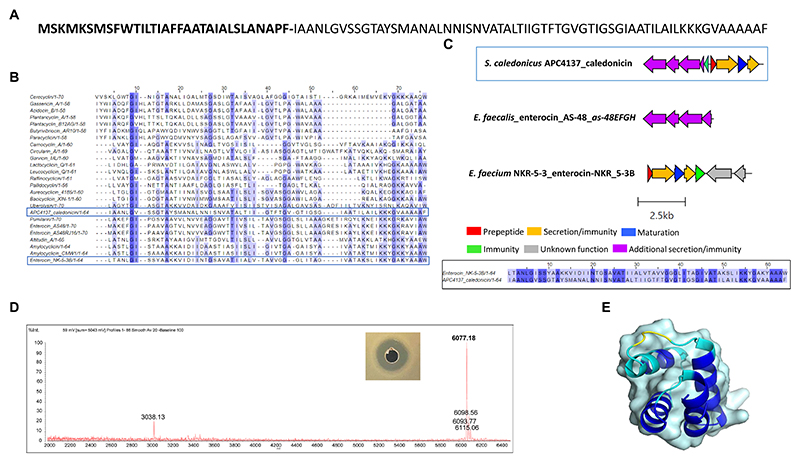
**(A)** Amino acid sequence of caledonicin with cut site distinguishing leader (bold) and core peptide identified **(B)** Multiple sequence alignment (MSA) of caledonicin bacteriocin from *S. caledonicus* APC 4137 and all known circular bacteriocins. Most closely related bacteriocin to caledonicin based on pairwise alignment of core peptide was enterocin-NKR-5-3B (39.1%) (blue boxes) **(C)** Operon alignment of caledonicin identified from APC 4137 with enterocin-NKR-53B operon (Genbank accession: LC068607.1), and pairwise alignment of caledonicin and enterocin-NKR-5-3B with conservation parameters set to 80% (black box). **(D)** Antimicrobial activity from partial purification elution in 70% IPA, 0.1% TFA against L. lactis subsp. cremoris HP and corresponding MALDI TOF mass spectrometry profile of 6,077.18 Da, where predicted mass calculated as 6,077.1 Da (Na+ adduct ion = 6,098.56Da; K+ adduct ion = 6,115.06 Da and potential oxidised peptide = 6,093.77Da). **(E)** Predicted structure of caledonicin as determined by Collabfold and viewed with PyMol v3.0 with extension to colour based on confidence where dark blue regions indicate high confidence (pLDDT > 90).

Following *in silico* analysis of the whole-genome sequencing, a putative novel circular bacteriocin from *S. caledonicus*, APC 4137, was identified by BAGEL4. The circular bacteriocin core peptide was found to be most closely related to enterocin-NKR-5-3B following pairwise alignment (39.1% identity) (Figures 7B,C), and the operons of these two bacteriocins were aligned using the clinker tool on CAGECAT (Gilchrist and Chooi, 2021).

Operon alignment revealed the caledonicin operon to possess all the genes required for circular bacteriocin synthesis as observed in enterocin-NKR-5-3B operon (Figure 7C), with the addition of five genes identified by BAGEL4 upstream from the prepeptide gene. Four of these five genes correspond to an ABC transporter permease, ATP-binding protein, putative efflux system protein, and one unknown function prediction, which have also been identified in enterocin-AS-48 (*as-48EFGH*) (Diaz et al., 2003) and carnocyclin A (*cclEFGH*) (van Belkum et al., 2010). Due to enterocin-AS-48 being the representative circular bacteriocin amongst this subclass, the *as-48EFGH* genes were aligned with these additional genes found using CAGECAT. CAGECAT demonstrated there was similarity between two of the four genes in the caledonicin operon with *as-48G* and *as-48H*. These genes were aligned in BLASTp with the corresponding caledonicin genes. The ATP-binding protein, *as-48G* shared 62.7% amino acid identity to the predicted ATP-binding protein in the caledonicin operon, while the membrane-spanning protein, *as-48H*, and the predicted permease protein shared 37.6% identity (Figure 7C). This shared percentage identity with *as-48G* and *as-48H* leads us to believe these four additional genes upstream of the caledonicin prepeptide correspond to an additional transporter system. Alignment of the enterocin-AS-48 biosynthetic operon, *as-48C1DD1*, was also performed but showed no percentage identity to the caledonicin operon and therefore was omitted from the study. Indeed, future assays would need to be carried out to confirm the effects of the additional proteins on bacteriocin synthesis and immunity, as has been conducted on the enterocin-AS-48 and carnocyclin A operons (Diaz et al., 2003; van Belkum et al., 2010). Interestingly, one gene was absent within the caledonicin operon when compared to enterocin-NKR-5-3B. This gene is annotated as the enterocin-NKR-5-3B immunity protein, *enkB4*. However, a gene was identified by BAGEL4 upstream of the prepeptide gene and downstream of the predicted transporter cluster discussed above. The amino acid sequence of this gene was put through the ExpasyProtparam online tool and found to be a small (67 amino acids), cationic (net charge +6), and hydrophobic protein (GRAVY index 1.243), all characteristics that correlate to the immunity protein of circular bacteriocins (Perez et al., 2018). Therefore, this gene is predicted to be the caledonicin immunity gene (Figure 7C). Based on these results, it can be assumed that the caledonicin operon displays amino acid identities across two circular bacteriocin operons, enterocin-AS-48 and enterocin-NKR-5-3B (Figure 7C). Following assessment of CFS, cell extract in 70% IPA, 0.1% TFA, and FT liquid against *L. lactis* HP and *M. luteus* APC 4061 (data not shown), the FT liquid was determined to be the extract exhibiting the best antimicrobial activity and was selected for purification of the bacteriocin. Caledonicin was subjected to partial purification, which was carried out on freeze−thaw samples previously shown to exhibit antimicrobial activity against *L. lactis* HP (data not shown). Partial purification was carried out using a 60-ml Strata C18-E column. The bacteriocin was eluted in concentrations of IPA between 50 and 70% and tested for activity via WDA, where the 70% IPA eluent was found to be active against *L. lactis* HP. This 70% IPA eluent was subjected to MALDI-TOF mass spectrometry, and a strong signal mass of 6077.18 Da was observed (Figure 7D), which correlated perfectly with the predicted mass of 6,077.1 Da. MALDI-TOF mass spectrometry also revealed masses potentially correlating to some oxidised caledonicin (+16 Da) and the sodium adduct ion (+22 Da). The doubly charged caledonicin ion (3,038.13 Da) was also observed (Figure 7D). The predicted structure of the circular bacteriocin was generated through ColabFold v1.5.5: AlphaFold2. This was followed by visualisation of the bacteriocin structure in the PyMOL Molecular Graphics System, Version 3.0. Percentage confidence of this structure is indicated by dark blue regions where pLDDT is >90 (Figure 7E).

#### Physicochemical assays

**Figure 8:**
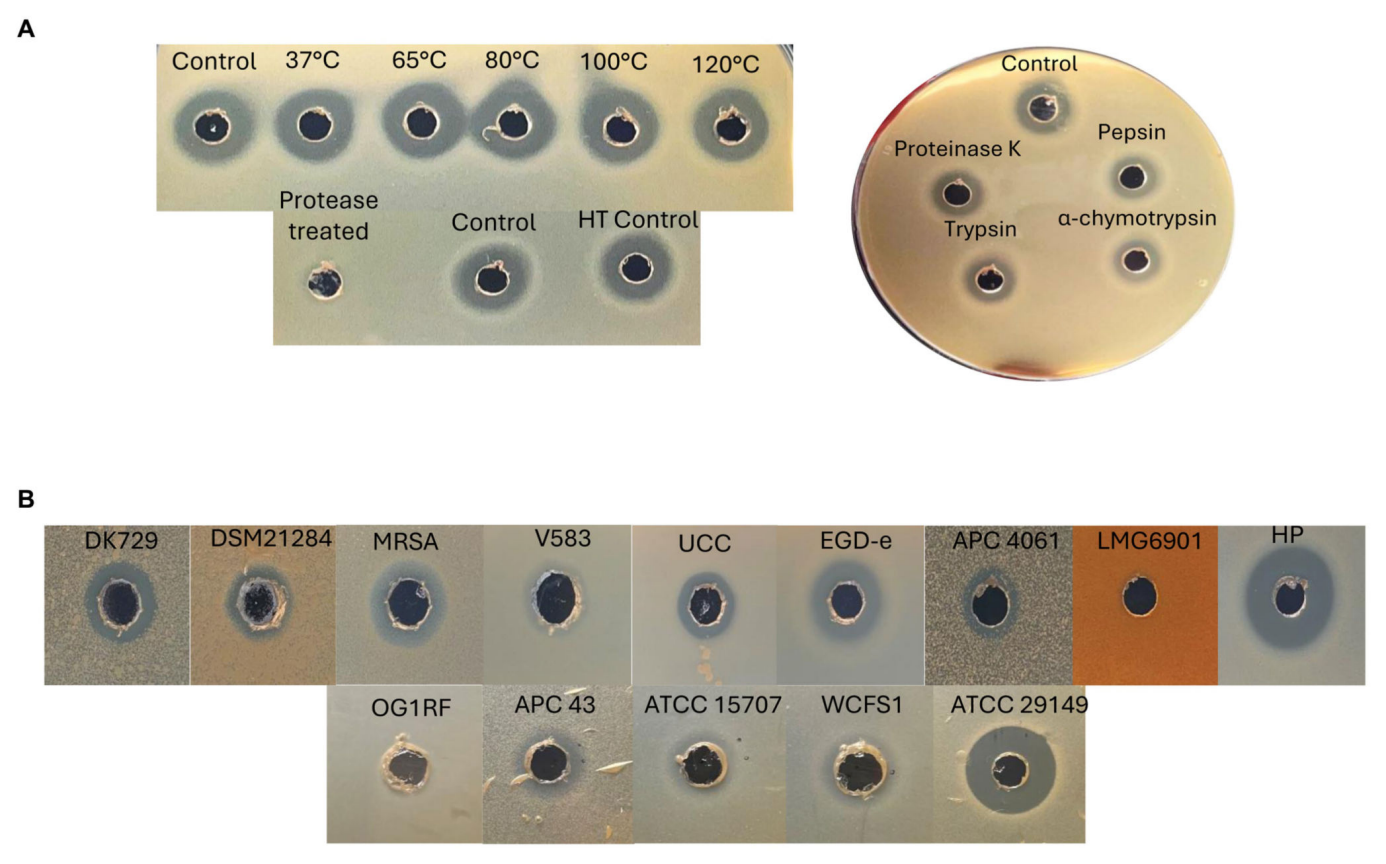
**(A)** Left: heat stability (top) of 70% IPA, 0.1% TFA caledonicin fraction, and protease degradation (bottom) of the same fraction following treatment with proteinase K at 20 mg/mL to demonstrate the antimicrobial’s proteinaceous nature. Right: protease sensitivity assay on 70% IPA and 0.1% TFA caledonicin fraction following protease treatment with proteinase K, pepsin, trypsin, and α-chymotrypsin at 100 μg/mL. **(B)** Spectrum of inhibition assays via WDA against Gram-positive indicators, including human gut-related SIHUMI strains. Prior to carrying out this assay, isopropanol from the 70% IPA and 0.1% TFA fraction was evaporated to concentrate the antimicrobial. Genus/species of strains listed in **(B)** are as follows: *S. pseudintermedius* DK729, *S. pseudintermedius* DSM 21284, MRSA DPC5645, *E. faecalis* V583, *L. innocua* UCC, *L. monocytogenes* EGD-e, *M. luteus* APC 4061, *L. delbrueckii* subsp. *bulgaricus* LMG6901, *L. lactis* subsp. *cremoris* HP. SIHUMI: *E. faecalis* OG1RF, *C. difficile* APC 43, *B. longum* ATCC 15707, *L. plantarum* WCFS1, and *M. gnavus* ATCC 29149.

Further tests were performed with the semi-purified active fractions, including exposure to proteases for 3 h, and heat stability at a range of temperatures (37, 65, 80, 100, and 120°C for 15 min). Protease treatments revealed the antimicrobial present in the active fraction to be proteinaceous in nature, as it was degraded completely by proteinase K at a high concentration of 20 mg ml^−1^ compared to untreated controls. The fraction was also subjected to the proteases proteinase K, pepsin, trypsin, and α-chymotrypsin (at lower concentrations of 100 μg ml^−1^) and shown to be somewhat protease-stable since only a partial reduction in antimicrobial activity compared to the untreated control was observed (Figure 8A). Heat treatment of the fraction demonstrated the antimicrobial to be heat-stable at all temperatures tested, as would be expected for bacteriocins (Figure 8A). This is the first evidence of a circular bacteriocin produced by a *Staphylococcus* species.

#### Spectrum of inhibition of caledonicin

The antimicrobial activity of semi-purified caledonicin was assessed via WDA against all gram-positive indicators listed in Table 1A, in addition to a panel of four human gut strains with an added *C. difficile* strain to assess the spectrum of activity of caledonicin against relevant veterinary- and human-related pathogens (Table 1B). Antimicrobial activity was observed against all gram-positive indicators listed in Table 1A, excluding *L. bulgaricus* LMG 6901. The highest activity was observed against *L. lactis* HP, as expected given the sensitivity of this strain to other bacteriocins, including nisin and lacticin 3147, with inhibition of growth also observed for *M. luteus* APC 4061 and *L. innocua* UCC. Strong inhibitory zones were also noted against a food-associated pathogen, *L. monocytogenes* EGD-e. Inhibition of the veterinary-related *S. pseudintermedius* pathogens (Field et al., 2015), DK 729, and DSM 21284 strains was also observed, which was surprising given the poor activity observed by the producing strain in colony-based deferred antagonism assays (Table 4). This increased activity in the WDA could be due to the increased concentration of the bacteriocin from the producing strain. Further WDAs of this fraction were carried out on a panel of human gut isolates (Ríos Colombo et al., 2023). Results showed *M. gnavus* ATCC 29149 exhibited the most sensitivity to caledonicin; however, there was a slight inhibition of the growth of *Lactiplantibacillus plantarum* WCFS1, *Bifidobacterium longum* ATCC 15707, and the human gut pathogen *C. difficile* APC 43 (Figure 8B).

## Discussion

Despite the evidence of increased antibiotic resistance, the global consumption of antibiotics by humans and animals is expected to increase by 200% by the year 2030, if no immediate action is taken (Klein et al., 2018; Iriti et al., 2020).

Worryingly, there is potential for cross-contamination of AMR bacteria not only between livestock and humans but also within companion animal settings. Transfer of commensal bacteria from pets to humans has been shown to be a two-way system, where it was highlighted that dog ownership resulted in a higher diversity of bacteria on the skin (hands and forehead) of dog owners compared to those without dogs (Song et al., 2013). However, transmission of AMR bacteria between pets and humans can also occur as a result of general contact with the animal, such as petting, or physical trauma/injury (e.g., dog bite) (Caneschi et al., 2023). Another method of drug-resistant bacterial transfer is via transformation, conjugation, or transduction of bacterial DNA from one strain to another, from owner to pet, or vice versa (Rendle and Page, 2018). For example, Tóth and co-workers detected genes from bacterial species in canine saliva conferring resistance to 13 different classes of antibiotics and determined the bacteria harbouring these genes, and the genes themselves, are capable of transferring and establishing themselves in humans and their bacteriome (Tóth et al., 2022).

This two-way transfer, in combination with the ongoing threat and reported health risk of potential AMR and ARG transmission between owners and their pets, suggests a need for novel antimicrobials for use in both companion animals and humans. One such alternative is bacteriocins, ribosomally synthesised antimicrobial peptides (AMPs) produced by bacteria that target closely related strains (Field et al., 2015).

Bacteriocins exhibit potent antimicrobial activity, in particular against AMR pathogens, highlighting their potential as alternatives to antibiotics (Meade et al., 2020). While several different environmental niches have been investigated in the past for novel bacteriocins, for example, human and animal gut isolates, skin, and breast milk (Birri et al., 2010; Han et al., 2014; O’Sullivan et al., 2019; Angelopoulou et al., 2020; Sugrue et al., 2020; Wosinska et al., 2022; Uniacke-Lowe et al., 2023), to the best of our knowledge, none have involved canine sources, with the exception of one study that focused specifically on enterococci from canine faeces and their antimicrobial potential against veterinary-related pathogens. This study, however, did not aim to screen for novel bacteriocins but rather aimed to identify enterocin A, P, B, L50A, L50B, AS-48, and *bac31* amongst 160 enterococci isolates (Kubašová et al., 2020). With that in mind, a screening study was designed and implemented to identify putative novel bacteriocins from canine sources.

Over 5,000 bacterial isolates from five canines were tested for antibacterial activity against a standard sensitive indicator, *L. lactis* HP. From these initial results, colonies of different morphologies producing phenotypically different inhibitory spectra against the indicator strain were selected for further testing. The majority of antimicrobial-producing bacteria of all sites tested in this study were isolated from the gum, with a crossover of bacterial species found between this area and their skin and/or nose based on 16S rRNA sequencing and WGS results. This is understandably so, as dogs can transfer bacteria from different areas of their bodies due to their grooming behaviour. A total of 42 isolates across the five canines were selected for further testing of cross-immunity and 16S rRNA sequencing. Based on the results obtained, 22 bacterial isolates of different genera and species were identified and selected for WGS. More specifically, of the 22 WGS isolates, the most abundant bacteriocin producers were isolated from the mouth (14/22, 63.6%), followed by the nose (4/22, 18.2%), and finally, the axilla and ear (2/22 each, 9.1%). Notably, the ears proved to be a poor source of bacteriocin-producing strains, where the only antimicrobial producers from this site were found in two of five dogs (*Paenibacillus* sp. APC 4171 and *B. safensis* APC 4157). This low abundance at this site could be due to the erect shape of the dog’s ears, which participated in this study, a trait thought to prevent overgrowth of bacteria or yeast due to low moisture and increased air circulation (O’Neill et al., 2021).

These 22 genomes were screened for their bacteriocinogenic potential and the presence of antimicrobial resistance genes (ARGs) and virulence factors to determine their suitability for probiotic use. Results of ARG and virulence factor presence in the WGS isolates in this study show there are ARGs present in almost all isolates, except for *A. bowdenii* APC 4154 and *B. pumilus* APC 4140, based on parameters set in the CARD and VFDB databases. This suggests these two strains could potentially have probiotic potential for companion animals (Supplementary Table S2).

Upon further *in silico* and *in vitro* investigation, the number of isolates selected for in-depth analysis was reduced to eight following further assessment for duplications of strain/species. The European Food and Safety Authority Panel on Animal Health and Welfare (AHAW) reported in 2021 a target working group of bacterial pathogens for AMR in dogs and cats, including *Enterococcus faecalis, E. coli, K. pneumoniae, P. aeruginosa, S. pseudintermedius*, and *S. aureus* (Nielsen et al., 2021). Based on these reported target pathogens, antimicrobial-producing isolates from this study were tested for antibacterial activity against a range of strains from the species mentioned above and others, including *L. monocytogenes* and *Salmonella enterica* serovar Typhimurium.

A total of 14 putative novel bacteriocins were identified from eight WGS bacterial isolates via *in silico* mining for prepeptide bacteriocin genes using BAGEL4, antiSMASH7.0, or Artemis (Table 3), from all five canines. Of these 14 bacteriocins, one particular bacteriocin of interest was a circular bacteriocin from strain *S. caledonicus* APC 4137, a previously identified novel species also isolated from a canine source (Newstead et al., 2021); however, no bacteriocins have been identified from this species to date. Based on this novel circular bacteriocin (termed caledonicin) being the first identified from this bacterial species, further tests were carried out to characterise the antimicrobial further. Circular bacteriocins are class IIc bacteriocins, so named due to their N- to C-terminal covalent linkage resulting in the formation of a ring structure. While these bacteriocins are currently classified as unmodified, it has been suggested they be reclassified to the modified class I bacteriocin grouping due to their simple yet modified structure (Alvarez-Sieiro et al., 2016; Pérez-Ramos et al., 2021; Sugrue et al., 2024). To date, approximately 20 circular bacteriocins have been identified, with enterocin-AS-48 as the representative bacteriocin for this class (Galvez et al., 1986). While these peptides share very little amino acid sequence similarity amongst each other, with leader sequences ranging between 2 and 48 amino acids, they do share characteristics such as thermal stability and protease resistance. These peptides have been further distinguished into two subgroups (I and II) by Perez and co-workers, where subgroup I is highly cationic and has a general isoelectric point (pI) >9, while subgroup II is highly hydrophobic, and their pI is <7 (Perez et al., 2018). Operons of circular bacteriocins generally contain genes encoding (i) the prepeptide, (ii) a maturation enzyme (sporulation M protein), (iii) an ABC transporter consisting of an ATP-binding domain and transmembrane permease for transportation/immunity purposes, and (iv) a small, hydrophobic immunity protein (Perez et al., 2018). This study has expanded further this class of bacteriocins discovered to date, with the identification of four novel circular bacteriocins in this study via *in silico* analysis and semi-purification and characterisation of one of these four peptides. Given the success of identifying novel bacteriocin producers from aerobic environments from canines in this study, further studies should be conducted on other areas, such as the gut microbiome of these animals and other pets to tackle AMR.

We describe caledonicin, a novel circular bacteriocin from *S. caledonicus* APC 4137 comprising a 64 amino acid core peptide based on structural characterisation (Figure 7E). Partial purification of the antimicrobial and subsequent MALDI-TOF mass spectrometry confirmed the presence of the bacteriocin (6,077.18 Da) in the purified fraction (Figure 7D). Pairwise alignment and bacteriocin operon alignment to enterocin-NKR-5-3B were also performed. Caledonicin is a subgroup I, heat-stable, protease-stable, circular bacteriocin that most closely resembles enterocin-NKR-5-3B based on percentage identity of the core peptide. Interestingly, the bacterial strain, when tested against indicators of interest in overlay assays, only exhibited activity against *L. lactis* HP, *L. innocua*, and *M. luteus* (Table 4). However, following WDA with semi-purified bacteriocin, caledonicin was found to inhibit the growth of a range of gram-positive pathogens, including the MRSP pathogens *S. pseudintermedius* DK 729 and DSM 21284 and the food-related pathogen *L. monocytogenes* EGD-e. This observation highlights the importance of using a sensitive indicator strain when initially screening for antimicrobials. Of the five SIHUMI strains tested, *M. gnavus* ATCC 29149 exhibited the most sensitivity to caledonicin, followed by a slight inhibition of the growth of *Lb. plantarum* WCFS1, *B. longum* ATCC 15707, and *C. difficile* APC 43. While *M. gnavus* ATCC 29149 is considered a human gut commensal, it has been associated with gut-related disorders/diseases, including irritable bowel disease/syndrome (IBD/IBS) and Crohn’s disease (CD), when present in high abundance (Crost et al., 2023) (Figure 8B). This lack of activity from overlay assays in comparison to semi-purified bacteriocin could be due to low concentrations in a bacterial culture and greater activity when concentrated.

In summary, these results demonstrate the promising properties of caledonicin in terms of stability and antimicrobial activity and suggest that this peptide merits further investigation as a novel antimicrobial alternative for both human and veterinary applications.

## Conclusion

Resistance to antibiotics is a serious threat, and their indiscriminate use has led to management restrictions for humans and animals. Bacteriocins are peptides synthesised by bacteria that can kill or inhibit the growth of other bacteria, which makes them invaluable for food preservation and potential therapeutic applications. In this study, we carried out the first bacteriocin screening study involving bacterial strains from canines and identified 14 putative novel bacteriocins based on prepeptide analysis of 22 bacterial isolates using whole-genome sequencing. One of these bacteriocins, caledonicin, is the first novel bacteriocin identified and characterised from *S. caledonicus*. It appears that the microbiome of canines represents an as-yet untapped but rich source of bacteriocin-producing bacteria, and based on the number of hits for antimicrobial compounds identified in this study on a sample size of only five canines, these animals are a worthy niche that warrants further investigation for antibiotic alternatives in the fight against AMR pathogens.

## Supplementary Material

The Supplementary material for this article can be found online at: https://www.frontiersin.org/articles/10.3389/fmicb.2024.1470988/full#supplementary-material

Supplementary Tables

## Figures and Tables

**Table 1:** Bacterial indicator strains and their growth conditions used in this study.

Species/strain	Conditions
*Lactococcus lactis subsp. cremoris* HP	GM17, aerobic, 30°C
*Micrococcus luteus* APC 4061	BHI, aerobic, 37°C
*Staphylococcus pseudintermedius* DK 729	BHI, aerobic, 37°C
*Staphylococcus pseudintermedius* DSM 21284	BHI, aerobic, 37°C
*Listeria innocua* UCC	BHI, aerobic, 37°C
*Listeria monocytogenes* EGDe	BHI, aerobic, 37°C
*MRSA* DPC 5645	BHI, aerobic, 37°C
*Enterococcus faecalis* VRE V583	BHI, aerobic, 37°C
*Cronobacter sakazakii* DPC 6440	LB, aerobic/anaerobic, 37°C
*Salmonella enterica serovar typhymurium* UK1	LB, anaerobic/aerobic, 37°C
*Klebsiella pneumoniae* NCIMB 13218	LB, anaerobic/aerobic, 37°C
*Escherichia coli* ETEC K88f4	LB, anaerobic/aerobic, 37°C
*Escherichia coli* ETEC F18ab	LB, anaerobic/aerobic, 37°C
*Escherichia coli* K12 MG1655 (ATCC 47076)	LB, anaerobic/aerobic, 37°C
*Pseudomonas aeruginosa* PA01	BHI, aerobic, 30°C
**B: Species/strain**	**Conditions**
*Lactobacillus delbrueckii* subsp. *bulgaricus* LMG6901	MRS, anaerobic, 37°C
*Enterococcus faecalis* OG1RF	LYHBHI, anaerobic, 37°C
*Lactiplantibacillus plantarum* WCFS1 (NCIMB 8826)	LYHBHI, anaerobic, 37°C
*Bifidobacterium longum* ATCC 15707	LYHBHI, anaerobic, 37°C
*Mediterraneibacter gnavus* ATCC 29149	LYHBHI, anaerobic, 37°C
*Clostridioides difficile* APC 43 (ATCC 43255)	LYHBHI, anaerobic, 37°C

Strains listed in Table 1B above were only tested against the circular bacteriocin, caledonicin identified in this study.

ATCC, American Type Culture Collection; APC, APC Microbiome Ireland Culture Collection; DPC, Teagasc Culture Collection; LMG, Laboratorium voor Microbiologie, Universiteit Gent, Belgium; DSM, Leibniz Institute DSMZ-German Collection of Microorganisms and Cell Cultures; UCC, University College Cork, Cork, Ireland; NCIMB, National Collection of Industrial, Food, and Marine Bacteria; LB, Luria-Bertani; BHI, Brain Heart Infusion; LYHBHI, BHI medium supplemented with 0.5% yeast extract (Difco); 5 mg/L haemin, cellobiose (1 mg/mL; Sigma-Aldrich), maltose (1 mg/mL; Sigma-Aldrich), and cysteine (0.5 mg/mL; Sigma-Aldrich), GM17; M17 supplemented with 0.5% glucose.

**Table 2:** Bacteriocin prediction and their classes as annotated by BAGEL4 and antiSMASH7.0 for 22 whole-genome sequenced strains in this study.

Strain ID	Genus/species	Area (canine isolated from)	Bacteriocin type (class)	
			BAGEL4	antiSMASH7.0
APC 4130	*Bacillus cereus*	Mouth (C1)	Thiopeptide ×2 (Class I), Sactipeptide (Class Ic), LAP (Class I)	TOMM (Class I), Thiopeptide ×4 (Class I), LAP (Class I), Sactipeptide (Class Ic)
APC 4133	*Bacillus cereus*	Mouth (C1)	Thiopeptide ×2 (Class I), Sactipeptide (Class Ic), LAP (Class I)	RRE-containing (no class), TOMM (Class I), LAP (Class I), Thiopeptide ×3 (Class I), Sactipeptide (Class Ic)
APC 4136	*Staphylococcus pseudintermedius*	Mouth (C1)	LAP (Class I), non-pediocin-like (Class IId), Sactipeptide ×2 (Class Ic)	LAP (Class I), Circular bacteriocin (Class IIc), non- pediocin-like (Class IId), TOMM (Class I)
APC 4137	*Staphylococcus caledonicus*	Mouth (C1)	Sactipeptide (Class Ic), Circular bacteriocin (Class IIc)	—
APC 4140	*Bacillus pumilus*	Mouth (C1)	Circular bacteriocin (Class IIc), Sactipeptide ×2 (Class Ic)	RRE-containing (no class), Sactipeptide (Class Ic)
APC 4145	*Staphylococcus warneri*	Mouth (C1)	Non-pediocin-like (Class IId), Sactipeptide (Class Ic)	Non-pediocin-like (Class IId)
APC 4147	*Staphylococcus caledonicus*	Mouth (C1)	Sactipeptide (Class Ic), Circular bacteriocin (Class IIc)	Circular bacteriocin (Class IIc)
APC 4148	*Bacillus cereus*	Mouth (C1)	Thiopeptide ×2 (Class I), Sactipeptide (Class Ic)	Thiopeptide ×3 (Class I), Sactipeptide (Class Ic), LAP (Class I), TOMM (Class I)
APC 4149	*Bacillus cereus*	Axilla (C1)	Thiopeptide (Class I), Sactipeptide ×2 (Class Ic), LAP (Class I)	Thiopeptide ×3 (Class I), TOMM (Class I), Sactipeptide (Class Ic), LAP (Class I)
APC 4152	*Staphylococcus pseudintermedius*	Mouth (C2)	LAP (Class I), circular bacteriocin (class IIc), Sactipeptide ×2 (Class Ic)	LAP (Class I), circular bacteriocin ×2 (Class IIc), TOMM (Class I)
APC 4153	*Staphylococcus pseudintermedius*	Mouth (C2)	LAP (Class I), circular bacteriocin (Class IIc), Sactipeptide × (Class Ic)	LAP (Class I), Circular bacteriocin ×2 (Class IIc), TOMM (Class I)
APC 4154	*Actinomyces bowdenii*	Mouth (C2)	Thiopeptide (Class I), Sactipeptide (Class Ic)	Thiopeptide ×2 (Class I)
APC 4156	*Staphylococcus pseudintermedius*	Nose (C2)	LAP (Class I), Circular bacteriocin (Class IIc), Sactipeptide ×2 (Class Ic)	LAP (Class I), Circular bacteriocin ×2 (Class IIc), TOMM (Class I)
APC 4157	*Bacillus safensis*	Ear (C2)	UviB (×2), LAP (Class I), Circular bacteriocin (Class IIc), Sactipeptide (Class Ic)	LAP (Class I), Circular bacteriocin (Class IIc)
APC 4158	*Actinomyces bowdenii*	Mouth (C3)	Thiopeptide (Class I), Sactipeptide (Class Ic)	Thiopeptide (Class I)
APC 4160	*Bacillus cereus*	Nose (C3)	LAP (Class I), Sactipeptide ×2 (Class Ic), Thiopeptides ×2 (Class I),	LAP (Class I), Thiopeptide ×3 (Class I), TOMM (Class I), Sactipeptide (Class Ic)
APC 4161	*Staphylococcus pseudintermedius*	Nose (C3)	LAP (Class I), Circular bacteriocin (Class IIc), Sactipeptide (Class Ic)	LAP (Class I), Circular bacteriocin ×2 (Class IIc), TOMM (Class I)
APC 4163	*Staphylococcus pseudintermedius*	Nose (C3)	LAP (Class I), Circular bacteriocin (Class IIc), Sactipeptide (Class Ic)	LAP (Class I), Circular bacteriocin ×2 (Class IIc), TOMM (Class I)
APC 4164	*Bacillus cereus*	Axilla (3)	LAP (Class I), Sactipeptide ×2 (Class Ic), Thiopeptides ×2 (Class I)	LAP (Class I), Sactipeptide (Class Ic) Thiopeptide ×3 (Class I), TOMM (Class I)
APC 4166	*Bacillus cereus*	Mouth (C4)	LAP (Class I), Sactipeptide ×2 (Class Ic), Thiopeptides (×2)	LAP (Class I), Sactipeptide (Class Ic) Thiopeptide ×3 (Class I), TOMM (Class I)
APC 4170	*Staphylococcus pseudintermedius*	Mouth (C5)	LAP (Class I), Circular bacteriocin (class IIc), Sactipeptide ×2 (Class Ic)	LAP (Class I), Circular bacteriocin ×2 (Class IIc), thiopeptide (Class I)
APC 4171	*Paenibacillus* sp.	Ear (C5)	Lasso peptide (Class I), Lanthipeptide ×2 (Class Ia), Sactipeptide ×4 (Class Ic)	Lasso peptide (Class I), Lanthipeptide ×2 (Class Ia)

**Table 3:** Putative novel bacteriocins predicted by *in silico* genome mining of WGS bacterial isolates from this study.

Strain ID	Genus/ species	Area	Bacteriocin	AA sequence (leader and core)	Predicted molecular weight (Da)	Identified by
APC 4154	*Actinomyces bowdenii*	Mouth (C2)	1. Arthropod defensin (×2); 2. Invert defensin containing domain; 3. Invert defensin containing domain	1. **MDKFTRRTADLASNDANKALNSETHTPLENAE**GFGCPFSAYECDRH CTSKGYRGGYCRGFVRQTCACY (×2); 2. **MPRFVRRSTALADATFKQAL HSETHAPTEGA**EYNCPTDEAPCDRHCRYSGYRGGYCGGMLKASCYCY (×1); 3. **MKKEMHMEIFSRRSRSLSDSRFNDTINSETRSPLETSE**HLSCPFNEHQCYKYCL SKGYRGGYCGGLAFAICRCY (×1)	1. 4044.50 2. 4087.50 3. 4106.60	Artemis
			*Thiopeptide	MNNVIDFAAIEISDLIEDAVDGGELPSQVMAASTTTSGCACSSCSSTCS	ND	BAGEL4 and antiSMASH7.0
APC 4170	*Staphylococcus pseudintermedius*	Mouth (C5)	Circular bacteriocin	**MLN**LHKKIAWTGVKGSAVTSFVSALATGSDIWAALTVAGIAFGGGVGTAIAAIG RATVVKFIKRWGVKKAAAW	7051.36	antiSMASH7.0
APC 4145	*Staphylococcus warneri*	Mouth (C1)	Lasso peptide	**MTESDFKKYNQLVGDRQLPGDVAQRT**GHNGVLSDDLMTNKIHYRKSEFSFCL	2994.41	Artemis
APC 4137	*Staphylococcus caledonicus*	Mouth (C1)	Circular bacteriocin (caledonicin)	**MSKMKSMSFWTILTIAFFAATAIALSLANAPF**IAANLGVSSGTAYSMANALNNISN VATALTIIGTFTGVGTIGSGIAATILAILKKKGVAAAAAF	6077.1	BAGEL4
APC 4140	*Bacillus pumilus*	Mouth (C1)	Circular bacteriocin	**MRASLILDHLNLSKFESIILAGFFATAALIGITLN**IGLIADFFGIKIAADWYRQLTD WLAAGGSLTTFAAIVMGVTLPAWLAAAATALGAYAA	5940.96	BAGEL4
APC 4157	*Bacillus safensis*	Ear (C2)	Circular bacteriocin	**1: MMK**VKKLGLISLLLFASTMASLVVSNGSSVATVITSVGVFFGVGIISSGVAAAIL AVLKKQGKAKAAAF **2: MTKATDSKFYALLSLSLLAVTLVALVIGNGSL**IAANLG VSTATAATVVNFLDTWSS VATVITIVGVFTGVGTISSGVAATILAILKKQGKAKAAAF	1: N/A 2: 6238.3	BAGEL4 and antiSMASH7.0
APC 4133	*Bacillus cereus*	Mouth (C1)	*Heterocycloanthracin family bacteriocin	MEGFIMNQFQQELQSLNLNDYQTGNVVYWDQQQSQYPYYYIQDDARRCGG CGGCGGRCGGCGGRCGGCAGRCGGCIGCAGCFSCFNCWNWWII	ND	antiSMASH7.0
			*Thiocillin family RiPP	**1:**MNKDLVKSVDNNTQALYIEEQVDATEF AGFTTAGSVATTSTLSSAGSCGGSFATGSSFSSAG **2**: MEELNENIYIEEQDDQLNEVAGTWGSASCFGSFSTFGGCAASASSTATASSAG **3:** MSKLSDSKPTDSAIYLEEQVELNEVAASLGSVSTFSSGSCPGSTVNTVSTASCQG	ND	antiSMASH7.0
APC 4171	*Paenibacillus* sp.	Ear (C5)	Lasso peptide	**MSKKEWQEPTIEVLDINQTM**AGKGWKQIDWVSDHDADLHNPS	2536.65	BAGEL4 and antiSMASH7.0
			Lanthipeptide	**MSNNQFDLDVQVSKNVGKIEPQ**VTSVFACTPGCITGPNCGSSECGTVPCGKTTSRLC	3317.95	BAGEL4 and antiSMASH7.0
			Lanthipeptide	**MKNQFDLDLQVAKDEVASKGVQPA**SGIICTPSCATGTLNCQVSLTFCKTC	2516.00	BAGEL4 and antiSMASH7.0

* Unable to predict the leader and core sequence. ND-mass was not determined due to intensive modifications and/or unable to distinguish the leader from the core peptide. Area of dog isolate was sourced is mentioned for each strain, with the specific canine in brackets.

**Table 4:** Spectrum of inhibition of putative novel bacteriocin-producing isolates from canine sources against indicators of interest via spot assays.

Strain↓/ indicator→	HP	APC 4061	DK729	DSM 21284	DPC 5645	V583	EGDe	UCC	UK1	NCIMB 13218	K88f4	F18ab	MG1655	PA01	DPC 6440
APC 4137 (*S. caledonicus*)	14.4 ± 3.3	4.1 ± 0.2	0	0	0	0	0	11.8 ± 0.6	0	0	0	0	0	0	0
APC 4140 (*B. pumilus*)	18.8 ± 1.1	11.5 ± 1.1	14.2 ± 5.9	11.7 ± 4.2	16.1 ± 4.1	0	0	0	0	0	4.1 ± 0.3	ND	0	0	0
APC 4145 (*S. warneri*)	5.4 ± 0.1	2.9 ± 0.3	0	0	0	0	0	0	0	0	0	0	0	0	0
APC 4157 (*B. safensis*)	20 ± 1.1	15 ± 0.8	20.8 ± 2.3	18 ± 0.4	19.5 ± 1.7	ND	4.8 ± 1.1	14.6 ± 0.4	0	0	4.8 ± 0.2	6.4 ± 4.1	0	0	0
APC 4170 (*S. pseudintermedius*)	6.9 ± 0.8	5.9 ± 0.2	0	0	0	3.6 ± 0.7	5.5 ± 0.8	5.9 ± 0.6	0	0	0	0	0	0	0
APC 4171 (*Paenibacillus* sp.)	18.8 ± 1.3	17.7 ± 1.3	11.7 ± 0.8	13.8 ± 0	12.7 ± 0.3	2.4 ± 0	10.7 ± 0.6	10.3 ± 0.7	15.9 ± 0.8	21.7 ± 0.7	17.7 ± 0.1	10.9 ± 0.4	20.3 ± 0.3	10.7 ± 0.6	16.9 ± 0.1
APC 4133 (*B. cereus*)	21.7 ± 1.1	19.3 ± 0.2	10.4 ± 1.1	8.7 ± 1.3	15 ± 0.4	12.9 ± 0.6	16 ± 1.9	17.1 ± 0.4	0	0	0	0	0	0	0
APC 4154 (*A. bowdenii*)	20.4 ± 1.6	6.7 ± 0.8	5.4 ± 0.4	3.7 ± 0.4	2.5 ± 0.1	5.2 ± 0.5	4.3 ± 0.8	4 ± 0.2	0	0	0	0	0	0	0

Genus and species of indicators listed in the top row are available in Table 1. ND, not determined.

## Data Availability

The datasets presented in this study can be found in online repositories. The names of the repository/repositories and accession number(s) can be found below: https://www.ncbi.nlm.nih.gov/, PRJNA1140517.

## Footnotes

1
https://www.ezbiocloud.net/tools/ani
